# *In vitro* Fab display: a cell-free system for IgG discovery

**DOI:** 10.1093/protein/gzu002

**Published:** 2014-02-28

**Authors:** Ryan L. Stafford, Marissa L. Matsumoto, Gang Yin, Qi Cai, Juan Jose Fung, Heather Stephenson, Avinash Gill, Monica You, Shwu-Hwa Lin, Willie D. Wang, Mary Rose Masikat, Xiaofan Li, Kalyani Penta, Alex R. Steiner, Ramesh Baliga, Christopher J. Murray, Christopher D. Thanos, Trevor J. Hallam, Aaron K. Sato

**Affiliations:** 1 Sutro Biopharma, Inc., 310 Utah Ave Suite 150, South San Francisco, CA 94080, USA; 2 Present address: Halozyme Therapeutics, Inc., New Molecular Entities, 11388 Sorrento Valley Road, San Diego, CA 92121, USA

**Keywords:** antibody reformatting, cell-free protein synthesis, Fab selections, ribosome display

## Abstract

Selection technologies such as ribosome display enable the rapid discovery of novel antibody fragments entirely *in vitro*. It has been assumed that the open nature of the cell-free reactions used in these technologies limits selections to single-chain protein fragments. We present a simple approach for the selection of multi-chain proteins, such as antibody Fab fragments, using ribosome display. Specifically, we show that a two-chain trastuzumab (Herceptin) Fab domain can be displayed in a format which tethers either the heavy or light chain to the ribosome while retaining functional antigen binding. Then, we constructed synthetic Fab HC and LC libraries and performed test selections against carcinoembryonic antigen (CEA) and vascular endothelial growth factor (VEGF). The Fab selection output was reformatted into full-length immunoglobulin Gs (IgGs) and directly expressed at high levels in an optimized cell-free system for immediate screening, purification and characterization. Several novel IgGs were identified using this cell-free platform that bind to purified CEA, CEA positive cells and VEGF.

## Introduction

A number of technologies have been developed which enable the discovery of antibodies and antibody fragments for therapeutic leads (Smith, 1985; Mattheakis *et al.*, 1994; Boder and Wittrup, 1997; Roberts and Szostak, 1997; Odegrip *et al.*, 2004; Reiersen *et al.*, 2005; Beerli *et al.*, 2008; Sumida *et al.*, 2009; Mao *et al.*, 2010; Sumida *et al.*, 2012). Although yeast and mammalian cells can express full-length immunoglobulin Gs (IgGs) (Chao *et al.*, 2006; Beerli *et al.*, 2008; Rakestraw *et al.*, 2009), most selections are usually performed using smaller antibody fragments. For instance, phage display is typically used for the selection of single-chain variable fragments (scFv) (McCafferty *et al.*, 1990) or Fab domains (Hoogenboom *et al.*, 1991). Phage and yeast display are very robust platforms, but they suffer from relatively low library diversities (∼10^6^–10^10^) due to limits of library transformation efficiencies. They are also hampered by the viability and toxicity of a certain fraction of the library inside living cells, or on the surface of viruses or cells. Thus, there has been a recent trend towards the development of technologies that enable selections to be performed entirely *in vitro* such as ribosome display (Hanes and Plückthun, 1997), CIS display (Odegrip *et al.*, 2004) and mRNA display (Fukuda *et al.*, 2006). These technologies overcome limitations in library transformation inefficiencies and the viability and toxicity issues of living systems. In theory, library sizes are only limited by the input mRNA/DNA quantity and the volume of the *in vitro* expression system (∼10^12–15^/ml). This assumes the library size is limited by the number of ribosomes and each protein expresses and folds well *in vitro*. These larger libraries are expected to facilitate discovery of relatively rare and higher affinity binders (Perelson and Oster, 1979; Ling, 2003).

The open nature of completely *in vitro* selection technologies has been thought to preclude the direct selection of multimeric proteins such as Fabs (Sumida *et al.*, 2009; Ponsel *et al.*, 2011)—which more closely resemble IgGs than scFvs—presumably because it has been thought that each protein chain must always maintain association with its own transcript. Phage and yeast display maintain the connection between multiple protein chains and their transcripts by sequestering them in the same living cell. This property has been mimicked *in vitro* by performing selections using artificial compartments (i.e. emulsion-based DNA display) to enable encapsulation of multiple protein fragments with their transcripts (Sumida *et al.*, 2009). However, the library sizes are then limited by the number of emulsions that can be practically produced (∼10^9^–10^10^ vesicles/ml) which is on the same order of magnitude as phage or yeast display. Recently, Fabs have actually been selected *in vitro* using mRNA display where the recovered heavy- and light-chain (HC and LC) pairs were re-coupled using gene-linking emulsion polymerase chain reaction (PCR) (Sumida *et al.*, 2012). In principle, single-chain Fab fragments (Hust *et al.*, 2007) could also be selected *in vitro*, but single-chain fragments are less preferable than two-chain Fabs since they are not as similar to natural multi-chain IgGs and have to be reformatted into a two-chain antibody after screening.

We present a simple approach for selecting large libraries of multimeric proteins such as Fabs that should apply to all completely *in vitro* selection technologies. This is achieved by simply maintaining the genotype–phenotype linkage for only one of the chains in a multi-chain protein. As a proof-of-concept, we initially show that the trastuzumab two-chain Fab can be displayed from ribosomes and selected from a crude cell-free reaction using the HER2/ErbB2 extracellular domain (ECD). We also show that synthetic naïve libraries of Fabs can be selected using ribosome display to find novel binders to carcinoembryonic antigen (CEA) (Hammarström, 1999) and vascular endothelial growth factor (VEGF) (Ferrara *et al.*, 2003). Moreover, we demonstrate that these Fabs can be reformatted to full-length IgGs which can be expressed at high levels and screened rapidly *in vitro* by exploiting open cell-free synthesis for IgG production (Yin *et al.*, 2012). This combined Fab display and cell-free expression platform provides a rapid way to discover lead IgGs from larger libraries of Fabs than is possible with other selection technologies.

## Materials and methods

### Construction of plasmids

The trastuzumab Fab HC and LC were cloned into the NcoI and HindIII sites of a pUC ribosome display vector containing the TolA spacer (same insert as pRDV, GenBank AY327136.1). The trastuzumab HC and LC pYD317 cell-free expression vectors have been described previously (Yin *et al.*, 2012). The reformatted full-length IgG HCs from the selection were cloned into the same pYD317 vector between NdeI and SalI restriction sites.

### Fab display proof-of-concept experiments

The HC display and expression templates were made by PCR of the trastuzumab HC Fab plasmid using either the T7B/TolAk primers (Dreier and Plückthun, 2011) or T7B/HER2_FAB_HC_STOP! RP (see Supplementary Table SI for primers), respectively. The LC display and expression templates were made by PCR of the trastuzumab Fab LC plasmid using either the T7B/TolAk primers or the T7B/Her2_FAB_LC_STOP! RP, respectively. All DNA templates were then purified with a Qiagen PCR purification kit. A total of 5 µl of a 3 : 1 mixture (based on mass) of the appropriate expression and display templates were added to 20 µl of pre-mixed PURExpress *in vitro* protein synthesis kit (NEB) containing 10 µl solution A, 7.5 µl solution B, 1 µl disulfide enhancer 1, 1 µl disulfide enhancer 2, 0.5 µl nuclease-free water (Ambion). The final concentration of the expression and display DNA templates were 300 and 100 ng/µl, respectively. The coupled transcription/translation reactions were allowed to proceed for 60 min at 30°C. The remaining selection was performed using standard protocols (Dreier and Plückthun, 2011) with 14 nM HER2/ErbB2 ECD (Creative Biomart) previously biotinylated. Reverse transcription (RT)-PCR was performed using a one-step Transcriptor RT-PCR kit (Roche) with T7B/TolAk primers. The RT-PCR product was sequenced using the TolA Internal RP. The experiment utilizing pre-purified LC protein (Supplementary Fig. S1) was performed by mixing 2.0 µg pre-transcribed HC Fab display mRNA (prepared using standard protocols) and 7.5 µg pre-purified LC protein (Yin *et al.*, 2012).

### HC library construction

Synthetic oligonucleotides containing degenerate codons (Supplementary Table SII) were dissolved to give 100 µM stocks. Primer sets were mixed to give similar degeneracy as Lee *et al*.: H1a For and H1b For were mixed at a 2 : 1 ratio to give H1 For mix; H2a For, H2b For and H2c For were mixed at a ratio of 1 : 2 : 0.1 to give H2 For mix; and H3a For and H3b For were mixed at a ratio of 1 : 1 to give H3 For mix. The complementary reverse primer sets were mixed separately at the same ratios. Four sets of PCR reactions were performed using (i) T7B/H1 Rev mix, (ii) H1 For mix/H2 Rev mix, (iii) H2 For mix/H3 Rev mix and (iv) H3F mix and Univ TolA RP. PCR was performed using 10 ng of the wild-type (wt) Fab HC ribosome display template and 0.5 µl of 100 µM primer stocks (∼3 × 10^13^ molecules) using 2× HF Phusion polymerase master mix (NEB). The fragments were gel purified and mixed at equimolar ratios (∼3.7 × 10^12^ molecules each) for overlapping PCR (18 × 50 µl reactions). The assembled library was then amplified using T7B/TolAk primers (48 × 50 µl reactions) which was then purified and concentrated using Zymo Research DNA Clean and Concentrator spin columns. Finally, an aliquot of the library was purified by gel extraction from a 1% agarose gel before the selection.

### LC library construction

Synthetic oligonucleotides containing degenerate codons (Supplementary Table SIII) were dissolved to give 100 µM stocks. The forward and reverse L3 oligos were mixed separately at equimolar ratios. For the shortest L1 loop-length sub-library, four sets of PCR reactions were performed using (i) T7B/CDR1-0 R, (ii) CDR1-0 F/CDR2 R, (iii) CDR2 F/CDR3 mix R and (iv) CDR3 mix F/Univ TolA RP. PCR reactions for the LC library fragments and assembly reactions were performed in the same manner as the HC library. The other L1 sub-libraries were made in a similar manner.

### Ribosome display selections

Library selections were performed essentially as described for the proof-of-concept experiments described above. For the first round of the HC selections, coupled transcription/translation reactions (10 × 25 µl each) were each performed using 7.5 µg pre-purified LC protein and 248 ng of the HC DNA library. For the first round of the LC selections, coupled transcription/translation reactions (10 × 25 µl each) were each performed using ∼200 ng of LC DNA library and 200 ng of the HC DNA expression template (made using T7B/HER2_FAB_HC_STOP! RP as in the proof of concept). For subsequent rounds, only two reactions were performed for the +Ag (25 µl each) and two reactions for the −Ag (25 µl each) using between 130 and 230 ng of each template DNA for each reaction. Selections were performed by using CEA (Meridian Life Sciences) or VEGF-121 (ACRObiosystems) previously biotinylated. Pre-selections with streptavidin-coated magnetic beads were performed for 1 h at 4°C for all rounds except the first. One-step RT-PCR was performed using primers specific for the Fab HC library: HER2 HCFAB Inner FP and HER2 HCFAB Inner RP or for the Fab LC library: HER2 LCFAB Inner FP and HER2 LC FAB Inner RP. Pooled sequencing was obtained using HER2 HCFAB Inner FP, HER2 LC FAB Inner FP, FabLC CenterSeq FP or FabLC CenterSeq RP. The mRNA from the LC selections was reverse transcribed and amplified using BsaI HERLC pYD FP/BsaI HERLC pYD RP and cloned into a modified pYD317 vector with BsaI sites inside of the NdeI and SalI sites. Following cloning, the BsaI sites are removed since BsaI is a type IIs endonuclease and the resulting vector reverts to the original NdeI and SalI flanking sites. Use of BsaI improves the ligation efficiency over NdeI and SalI.

### Overlapping PCR assembly of full-length IgG HCs

The output mRNA from the selection was amplified using Fab FP/RP and a one-step Transcriptor RT-PCR kit to create the DNA for the Fab fragment. The wt Fc fragment was created by PCR using Phusion polymerase and Fc FP/RP with the wt trastuzumab plasmid in pYD317. The Fab and Fc fragments were gel purified and an equimolar mixture was amplified using overlapping PCR with Phusion polymerase in the absence of primers for 10 cycles. Then, 5 µl of this crude reaction was used as a template for amplification using HC FP/RP for 25 cycles with Phusion polymerase and purified using a PCR spin column. The product was digested with NdeI and SalI, gel purified, ligated into pYD317 and transformed into DH5α cells. Colonies were picked, mini-prepped and sequenced to isolate individual clones.

### Small-scale expression of IgGs and enzyme-linked immunosorbent assay screening

Cell-free reactions were run essentially as described previously using DsbC, PDI and pre-purified trastuzumab LC protein (Yin *et al.*, 2012). Small-scale coupled transcription/translation was initiated by the addition of 5 µl of non-normalized mini-prepped DNA (∼50–150 ng/µl) to the cell-free mixture (30 µl final volume) and was incubated at 30°C for 6 h in a covered 96-well microtiter plate while shaking at 800 rpm using an Eppendorf Thermomixer R. The crude cell-free reactions were stored at 4°C until they were centrifuged at 5000 *g* for 5 min at 4°C. The supernatant was diluted into 500 µl PBST. A separate MaxiSorp plate was incubated overnight at 4°C with 100 µl of 5 µg/ml of CEA, HER2/ErbB2 ECD or streptavidin in phosphate-buffered saline (PBS). The antigen solution was removed and the plate was blocked with 2% bovine serum albumin (BSA) in PBST for 1 h at room temperature while shaking (500 rpm). Then, 100 µl of the diluted crude cell-free reactions were added to the wells and incubated at room temperate for 1 h with shaking. The plate was washed five times with PBST and 100 µl of 1 : 10 000 dilution of anti-human IgG (Fc specific)-peroxidase antibody conjugate (Sigma) in PBST was incubated for 1 h at room temperature with shaking. The plate was washed five times with PBST and developed with 100 µl 3,3′,5,5′-tetramethylbenzidine for 16 min before quenching with 100 µl 1 N sulfuric acid. Absorbances were measured using a SpectraMax plate reader. A mouse anti-VEGF positive control was used for the LC selections (Abcam) which was visualized with a secondary anti-mouse antibody-horseradish peroxidase conjugate (Sigma).

### Expression and purification of IgGs at larger scale

Cell-free reactions were performed on 3 or 20 ml scale according to the same protocols described for wt trastuzumab IgG (Yin *et al.*, 2012). The 20 ml crude cell-free reactions were diluted in 50 mM sodium phosphate, pH 7.3 and subsequently clarified via centrifugation. The IgGs were then captured by means of MabSelect SuRe (GE Lifesciences) chromatography, and further purified on Capto Adhere (GE Lifesciences) using manufacturer recommendations.

### Enzyme-linked immunosorbent assay titrations

The enzyme-linked immunosorbent assay (ELISA) titration with purified IgGs was performed in duplicate essentially as described for the crude IgGs described above. The purified IgGs were diluted in PBST to give a 3- or 5-fold dilution series for IgGs derived from the LC and HC selections, respectively.

### Surface plasmon resonance

For the IgGs derived from the Fab HC selections, a Biacore T100 instrument was used. Streptavidin (250 µg/ml final concentration after dilution) was immobilized to all four flow cells in 10 mM acetate pH 4.5 using standard EDC/NHS coupling onto a Series S Sensor Chip CM1. Biotinylated CEA was diluted to 20 µg/ml in the running buffer (10 mM HEPES pH 7.5, 150 mM NaCl, 0.5% BSA and 0.05% P2) and immobilized to the streptavidin on flow cells 2–4. A 2-fold dilution series of each IgG from ∼80 nM down to ∼1 nM was performed in duplicate. Each injection was performed for 180 s at 40 µl/min and dissociation was followed for 120 s. Regeneration was performed using 3 × 30 s injections of 3 M MgCl_2_ at 20 µl/min. The wt trastuzumab also produced in our cell-free extract showed no binding to the immobilized CEA.

All measurements for IgGs from the Fab LC selections were carried out on a Biacore T200 instrument. Biotinylated rhVEGF121 (AcroBiosystems #VE1-H4213) was directly immobilized using amine coupling chemistry by flowing a 1 µg/ml solution of rhVEGF121 in acetate buffer pH 5.0 over flow cells 2 and 4 of a Series S CM5 Sensor chip (GE Healthcare #BR-1005-30). Similarly, rhErbB2/Her2-Fc (R&D systems, #1129-ER) was directly immobilized on a separate Series S CM5 sensor chip. A 2-fold dilution series of each IgG starting at 125 nM down to 6.25 nM was injected at a flow rate of 50 µl/min over the rhVEGF121 and rhErbB2/Her2-Fc surfaces in separate runs to measure the binding affinities. Association and dissociation phases for the antibody–antigen interaction were followed for 180 and 600 s, respectively. Regeneration was carried out 2 × 30 s injections of 100 mM glycine pH 1.5. As controls, the binding of ranibizumab wt Fab (VEGF binder) and trastuzumab wt IgG (ErbB2/Her2 binder), both produced in our cell-free extract, were also measured which were consistent with the reported literature values.

### Caliper analysis

LabChip GXII (Caliper LifeSciences/Perkin Elmer) was used for analysis of purified IgG concentration and purity, using the Protein Express Chip assay. Assay reagents were prepared per manufacturer guidelines and ran using high sensitivity mode. Samples 2-B5, 2-D1, 2-G3 and 3-E6 were diluted in PBS, pH 7.4, 2- and 4-fold for analysis. Diluted samples (5 µl) were mixed with 7 µl Protein Express Sample Buffer using a 96-well PCR plate, denatured at 65°C for 10′ and mixed with 32 µl Milli-Q water. For quantification, a trastuzumab standard curve ranging from 500 to 10 µg/ml was prepared in an identical manner to the analyzed samples. The 96-well PCR plate was ran on the LabChip GXII and the samples were quantified using LabChip GX software v3.1 (Caliper LifeSciences/Perkin Elmer).

### Fluorescence-activated cell sorting-based cell binding assay

CEA-expressing MKN45 cells (Japanese Cancer Research Bank, Cat# 0254) and Her2 expressing SKBR3 cells (ATCC, Cat# HTB-30) were cultured in Dulbecco's modiﬁed Eagle's medium and Ham's F-12, 50/50 Mix medium (Mediatech, Cat# 15090CM) supplied with 10% fetal bovine serum (Thermo Scientific, Cat# SH30910.03/DEL), 1% GlutaMAX (Life technology, Cat# 35050-061) and 0.5% Pen/Strep (Mediatech, Cat# 30-001-CI). Cells were harvested using Cell Detachment Solution (BD Biosciences, Cat# 561527) and washed once with fluorescence-activated cell sorting (FACS) binding buffer (PBS supplied with 0.5% BSA and 0.1% sodium azide). The cells were then re-suspended with ice-cold FACS binding buffer. The antibody samples were serial diluted in a 96-well plate and 200 000 cells were added into each well. The plate was kept on ice for 1 h and washed twice with FACS binding buffer. Alexa647 labeled goat anti-human Fc antibody was added and incubated with the cells for 1 h on ice. After washing twice with FACS binding buffer, the cells were fixed with 2% paraformaldehyde (USB Corporation Cat# 19943) for 10 min, washed once again with FACS binding buffer. Samples were acquired using a Becton–Dickinson FACS Calibur flow cytometer-automated micro-sampler (Cytek Development Inc.) and analyzed using Flo Jo software (Treestar, Inc., Ashland, OR, USA). Binding *K*_D_s were calculated by using GraphPad Prism software. Non-linear regression analysis and a one site-specific binding parameter were used to calculate the binding *K*_D_.

## Results

### Testing the Fab display concept

**Fig. 1.**
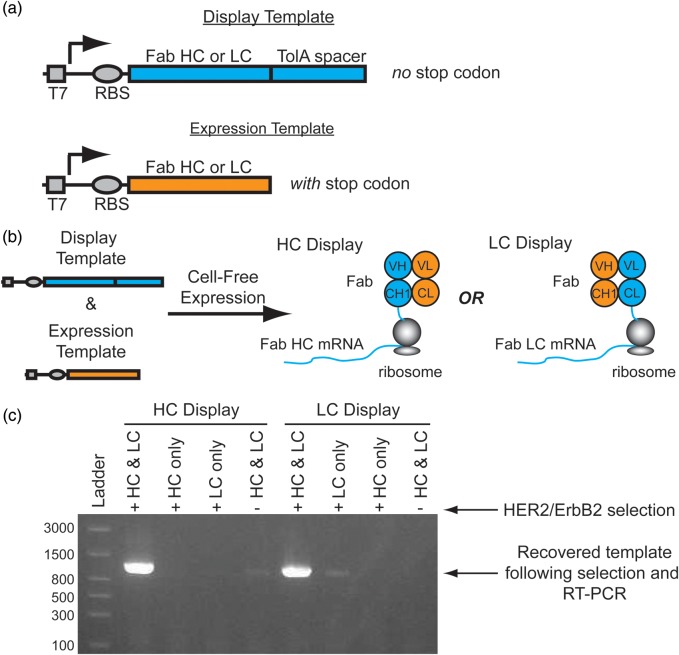
Conceptual overview of Fab display. (**a**) To display functional heterodimeric Fab domains from ribosomes two DNA templates are required for transcription: 1. A ‘Display Template’ lacking a stop codon at the end of a disordered TolA spacer, and 2. An ‘Expression Template’ with a stop codon. (**b**) A mixture of a display template and its complementary expression template co-expressed in a cell-free translation mixture enables the formation of a ternary complex of the functional Fab, ribosome and mRNA complex. For HC display, the HC display template is co-expressed with the LC expression template which enables the selection of a HC library. Alternatively, co-expression of the LC display template with the HC expression template enables LC display. (**c**) Co-expression of *both* the trastuzumab Fab HC and LC display and expression templates in either the HC or LC display format is required for significant recovery of the template following selection with Her2/ErbB2 ECD. This is consistent with the assembly of functional heterodimeric Fabs on stalled ribosomes as the models in (b) suggest. A reproducible, weak band is observable in the HC Display format when HC and LC are expressed in the absence of HER2/ErbB2 selection. This is presumably due to non-specific binding of the Fab-HC mRNA-ribosome complex. Similarly, a weak band is observable in the LC Display format when the LC only is displayed due to non-specific binding.

In principle, selecting a heterodimeric Fab domain by ribosome display can be performed using two templates: (i) a display template containing the Fab HC fused to a TolA spacer *without* a stop codon, and (ii) a complementary expression template containing the Fab LC *with* a stop codon (Fig. 1a). The lack of a stop codon at the end of the display template forces stalling of the ribosome at the end of the HC mRNA transcript enabling formation of the ternary protein-ribosome-mRNA complex. The TolA spacer is a standard unstructured domain used in ribosome display which allows the nascent Fab HC to project from the exit tunnel and fold outside of the ribosome (Dreier and Plückthun, 2011). The LC expression template contains a stop codon at the end of the transcript and lacks the TolA spacer since it is not intended to be displayed directly from the ribosome nor linked to its own mRNA transcript. Instead, co-expression of the LC enables heterodimerization with the displayed HC on the ribosome to yield the complete Fab-ribosome-HC mRNA ternary complex (Fig. 1b). Alternatively, the Fab can be linked to the LC mRNA by expressing the LC from the display template and its complementary HC from an expression template. In this manner, libraries of either the HC or LC can be screened in turn.

To demonstrate this Fab display concept, we cloned the Fab HC and LC domains from trastuzumab into separate ribosome display vectors containing the same T7 promoter, RBS and TolA spacer. PCR was used to generate the display and expression templates for both the HC and LC domains. A mixture of the HC display template and the LC expression template was added to a cell-free expression system to enable simultaneous coupled transcription/translation from both templates. Following standard ribosome display selection protocols (Dreier and Plückthun, 2011) with biotinylated HER2/ErbB2 ECD, a robust DNA band was recovered by RT-PCR corresponding to the display transcript (confirmed by DNA sequencing) (Fig. 1c, left side). Neither the HC display template nor the LC expression template alone yielded any HC transcript, suggesting that both templates are necessary for transcript recovery following selection. The negative control experiment mixing both the HC display and LC expression templates and performing the selection in the absence of HER2/ErbB2 also failed to yield a significant band on a gel. We then performed the analogous set of experiments, using the LC display template and the HC expression template, which yielded similar results (Fig. 1c, right side). Since the trastuzumab LC can be expressed and purified in isolation (Yin *et al.*, 2012), we also showed that mixing the pre-purified LC protein with the HC display mRNA in a cell-free expression system enables display and selection of Fab domains on ribosomes (Supplementary Fig. S1). Altogether, these experiments suggest functional, heterodimeric Fab domains can be displayed from the ribosome with either the HC or LC mRNA connected to its respective HC or LC protein.

### Construction of a naïve, synthetic Fab HC display library

To demonstrate the ability of Fab display to select for novel antibodies, we constructed a validated library on the trastuzumab Fab HC domain which was used in our initial proof-of-concept experiments. Lee *et al.* (2004) have described a suitable Fab library based on the trastuzumab scaffold in which HC and LC libraries are selected independently by phage display. They performed iterative experimentation limiting the diversity on CDR H1 and H2 and controlled randomization of CDR H3 using XYZ codons over a length diversity of 7–19 amino acids. They ultimately used XYZ codons, which contain unequal ratios of the four nucleotides, to match the natural CDR amino acid diversity better than NNK codons. Accordingly, we designed a HC library using the same diversity on CDR H1 and H2. For simplicity, we limited our library's CDR3 to a single length of 11 amino acids (Lee *et al.*'s most common length) and randomized 8 of the 11 CDR H3 residues with NNK instead of XYZ codons. Mixtures of oligos were used to enable specific amino acid mixtures not accessible by simple degenerate codons such as NNK. For instance, a 1 : 1 mixture of Phe and Met was desired at position H100C of the HC CDR3, so two oligos were mixed at a 1 : 1 ratio with TTT and ATG codons at H100C since Phe and Met cannot be coded exclusively with a single degenerate codon.

**Fig. 2.**
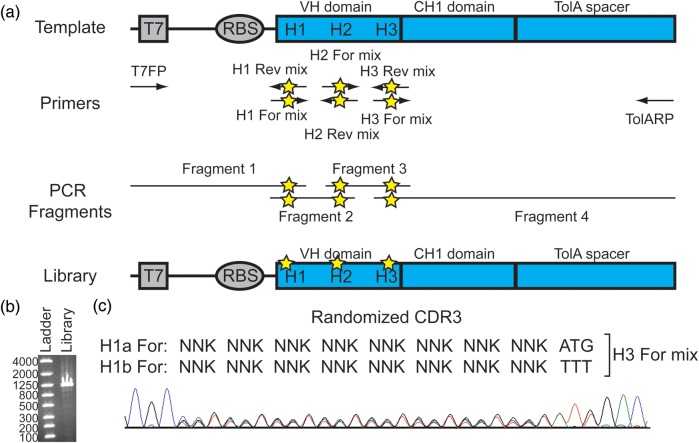
Fab HC library construction and characterization. (**a**) The template plasmid containing the trastuzumab HC Fab is used to generate four separate PCR fragments using four sets of primers. The residues in CDR H1, CDR H2 and CDR H3 are randomized using degenerate base pairs (stars) in the H1-H3 primer mixes. The final library is constructed by overlapping PCR using these individual fragments. (**b**) The final crude assembled library consists of a single band of the desired size (1103 bps) on an agarose gel. (**c**) Pooled sequencing of the final naïve library shows the intended degeneracy in the coding region for CDR3. See Supplemental Fig. S2 for pooled sequencing of the coding regions of CDR1 and CDR2.

To generate the Fab HC library, we employed an overlapping PCR protocol (Fig. 2a). Four sets of primers were designed to give four different PCR fragments containing the designed nucleotide diversity at each of the CDRs of the HC. The ends of each PCR fragment are complementary to the next fragment which can all be assembled together by overlapping PCR to produce the complete Fab display library (Fig. 2b). Pooled sequencing (Pelletier *et al.*, 1999; Nurpeisov *et al.*, 2003; Meng *et al.*, 2007) of the final library confirms the intended degeneracy at each position (Fig. 2c and Supplementary Fig. S2). Since Fab display is performed entirely *in vitro*, the final assembled PCR product can be used directly. This feature maintains the library size by eliminating losses due to *in vivo* viability of individual library members or transformation and ligation inefficiencies.

### Performing a Fab display selection from a naïve Fab HC library

**Fig. 3.**
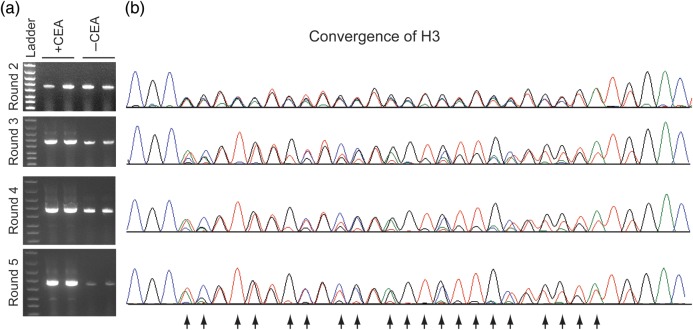
Analytical data collected to monitor the HC selection. (**a**) Selections were performed in the presence or absence of CEA in duplicate. By Round 3, there is a clear difference in the amount of DNA recovered in the presence versus the absence of CEA following RT-PCR. This is consistent with enrichment of CEA binders. By Round 5, the population is almost entirely enriched for CEA binders. The ladder is the same as in Fig. 2b. (**b**) Pooled sequencing centered on CDR H3 of the output DNA following RT-PCR after each selection round indicates convergence to specific bases or mixtures of bases (arrows) from the original degenerate codons. The convergence closely tracks the enrichment observed in the gel analysis in (a). See Supplemental Fig. S3 for pooled sequencing analysis of CDR1 and CDR2 during selection.

For the first selection round, 2.5 µg of the Fab HC library was used as the display template in a cell-free expression system capping the upper diversity limit at ∼2 × 10^12^ total DNA molecules. For simplicity, the purified LC protein was used rather than expressing the LC from a DNA template. The naïve Fab library was then selected for binding to CEA. Beginning at the second round, a control selection was performed in the absence of CEA which enables facile monitoring of enrichment of CEA binders by running an agarose gel of the RT-PCR product after selection (Fig. 3a). By round 3, there is a clear increase in the recovery of DNA following RT-PCR in the selections performed in the presence versus the absence of CEA indicating an increase in CEA-binding Fabs during the selection. Pooled sequencing of the output RT-PCR DNA from each round also shows many of the degenerate positions from the naïve library have converged to just one or two individual bases (Fig. 3b and Supplementary Fig. S3). This convergence starts at round 3 throughout each CDR and tracks well with the enrichment as assessed by RT-PCR. This suggests that the pooled sequences reflect the underlying sequences of the dominant CEA-binding Fabs. Five rounds of selection were performed to ensure that the majority of the selected Fabs would bind to CEA before screening individual clones.

### Screening IgGs for CEA binding and expression from a Fab HC selection

**Fig. 4.**
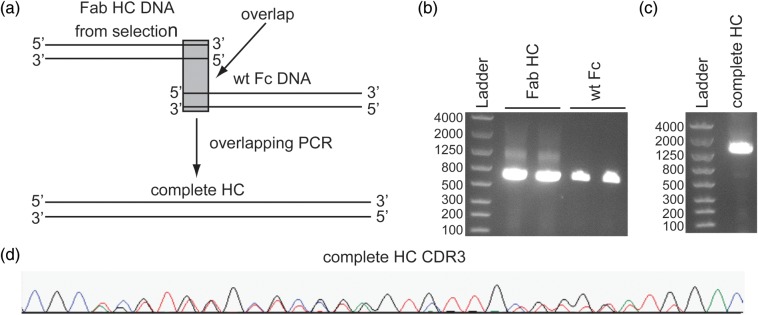
Reformatting selected Fab HCs to IgGs by overlapping PCR. (**a**) Overlapping PCR was used to generate the complete HC for subsequent IgG expression. (**b**) The Fab HC DNA was produced by RT-PCR of the output mRNA from Round 5 of the selection (expected size ∼670 bps). The wt Fc DNA including the hinge region and an overlapping segment of ∼30 bps into the CH1 domain was produced by PCR from a wt plasmid (expected size ∼700 bps). (**c**) The final assembled complete HC DNA is the main product and the correct size (expected size ∼1353 bps). (**d**) As desired, pooled sequencing of the assembled complete HC DNA does not indicate any significant differences from before assembly in the ratios of nucleotides observed throughout CDR3 (compared to Round 5, Fig. 3b).

Before subsequent screening and expression, the selected Fabs were reformatted into IgGs using overlapping PCR (Fig. 4). Pooled sequencing of the complete HC suggests that the overlapping PCR procedure does not substantially alter the distribution of the selected clones (compare Round 5 of Fig. 3b with Fig. 4d). The reformatted complete HCs were sub-cloned into a vector suitable for cell-free expression and 39 individual IgG HC expression vectors were isolated and sequenced (Supplementary Fig. S4). Sequencing from the 39 clones indicated that there were 24 unique CDR combinations though most appeared to be mixtures of the same set of CDRs as has been seen in other ribosome display selections (Larman *et al.*, 2012). In total, there were 8 unique H1, 10 H2 and 8 H3 CDRs. The apparent CDR shuffling presumably occurs through PCR-mediated recombination between the selection steps (Meyerhans *et al.*, 1990), but a template-switching process involving reverse transcriptase (Luo and Taylor, 1990) or some other mechanism cannot be ruled out. Most of the clones had additional mutations outside of the CDRs as is usually observed in ribosome display selections (Hanes and Plückthun, 1997). Thus, even clones with the same CDR combinations usually have unique overall sequences.

**Fig. 5.**
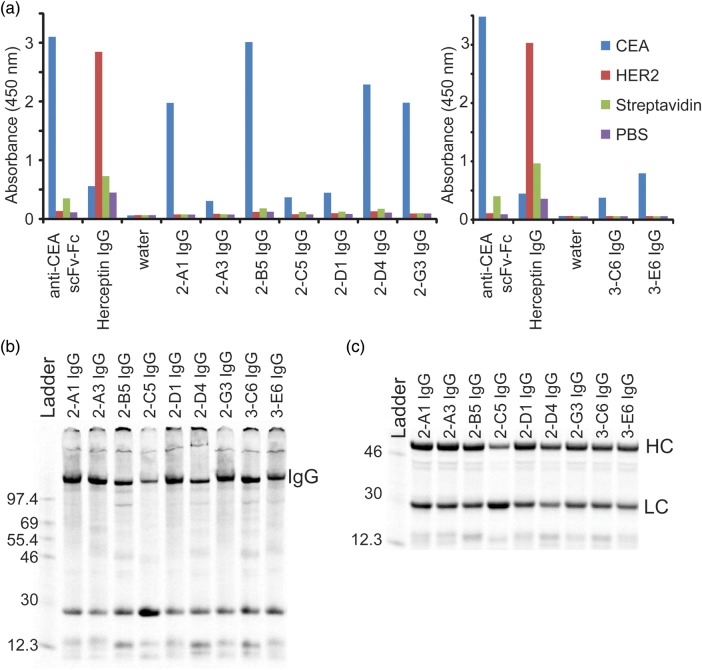
Screening and expression of IgGs with selected HCs. (**a**) IgGs from the selection were expressed on small scale (30 µl) in cell-free extract and screened by ELISA to find leads that were specific for CEA over HER2/ErbB2, streptavidin and a PBS control. Positive controls show that an anti-CEA scFv-Fc is specific for CEA and the trastuzumab IgG is specific for HER2/ErbB2. Experiments performed on separate days are presented in separate charts. (**b**) A non-reducing SDS–PAGE gel shows significant expression of each selected IgG at the correct molecular weight for the fully assembled product. Proteins are labeled with ^14^C-Leu. (**c**) A reducing SDS–PAGE gel shows good expression of the majority of the reformatted complete HC and LC. Proteins are labeled with ^14^C-Leu. A ^14^C-labeled protein ladder (in kDa) is shown on the left lanes of the gels in (b) and (c).

Cell-free expression was performed on small scale (30 µl) with each isolated clone in the presence of pre-purified LC protein and the crude reactions were screened by ELISA for IgGs that bound specifically to CEA (Fig. 5a). A scFv-Fc derivative of MFE-23, a known CEA binder (Chester *et al.*, 1994), and the trastuzumab IgG were also expressed and screened to ensure the activity of the cell-free system and that CEA and HER2/ErbB2 antigens were folded. A positive ELISA signal for CEA binding was observed for 28 of the 39 clones and none showed any significant binding to the original Her2/ErbB2 antigen, streptavidin or an uncoated well (i.e. a no antigen PBS control). The ELISA signals vary substantially for all the crude samples, but the differences are not expected to be due to the variation in binding affinity alone, as there are many other potential factors like expression levels, folding efficiency, etc. Thus, the ELISA acts as a preliminary screen for lead IgGs that specifically bind to the intended antigen and express reasonably well in cell-free extract to give functional protein. Accordingly, a total of nine individual IgGs covering a range of different sequences with strong intensity of the ELISA signal were chosen for moderate scale-up (3 ml cell-free reaction) (Fig. 5a). Cell-free reactions were performed in the presence of ^14^C-Leu for these select IgGs and most show good expression levels (Fig. 5b and c). A non-reducing sodium dodecyl sulfate–polyacrylamide gel electrophoresis (SDS–PAGE) gel shows that all reactions yield a band consistent with the size of the correctly assembled IgG (Fig. 5b). Moreover, a reducing gel of the same material shows each IgG can be broken down into its HC and LC components (Fig. 5c).

### IgG characterization from Fab HC selection

**Fig. 6.**
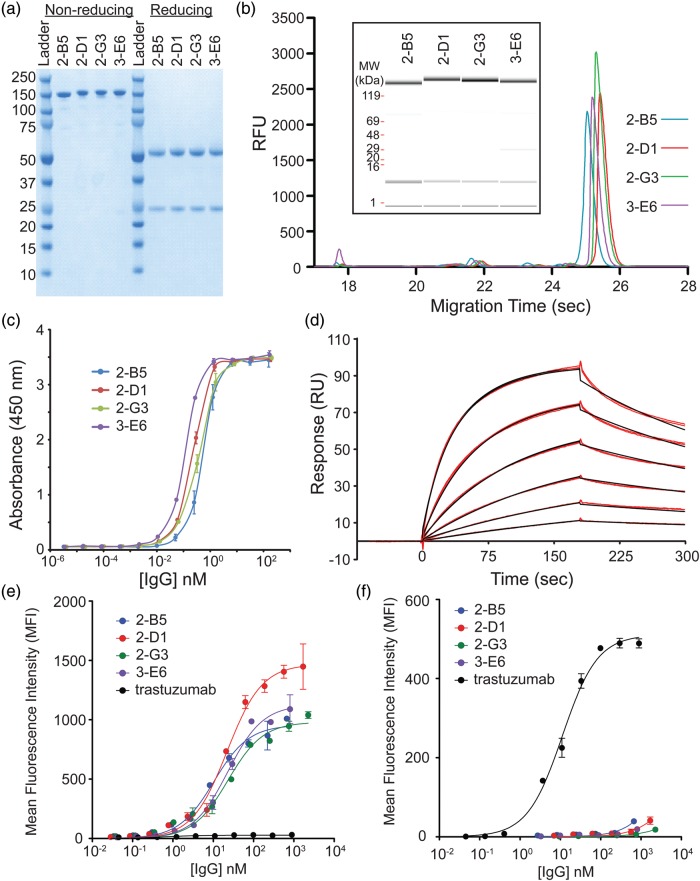
Characterization of purified IgGs. (**a**) The final purified anti-CEA IgGs run predominantly as a single band on a non-reducing SDS–PAGE gel consistent with the full-length IgG. Under reducing conditions, bands consistent with the HC and LC fragments are observed. (**b**) Caliper analysis reveals the high purity of the final IgGs. The inset shows the same sample traces where the amount of protein is indicated by the band intensity. (**c**) An ELISA of the purified anti-CEA IgGs have sub-nanomolar EC_50_ values for CEA. (**d**) A sample Biacore trace (red lines) for the 2-G3 anti-CEA IgG and global fit to a bivalent binding model (black lines). Binding was measured in duplicate at 1.3, 2.5, 5.1, 10.1, 20.3 and 40.5 nM over three separate channels. A bivalent model fit the data better than simple 1 : 1 binding as expected for a bivalent IgG. (**e**) The reformatted IgGs show apparent high affinities on MKN45 cells which express high levels of CEA, while the wt trastuzumab (expressed in CHO) IgG does not. (**f**) The wt trastuzumab IgG (expressed in CHO) binds to the ErbB2/HER2 expressing SKBR3 cells, but the selected CEA-specific IgGs do not.

Four IgGs with unique CDRs were scaled up using 20 ml cell-free reactions for additional characterization. Total crude yields were quantified by incorporation of ^14^C-Leu into a small sample (100 µl) of the total 20 ml cell-free (Supplementary Fig. S5). Yields were comparable to a wt trastuzumab IgG control (between ∼200 and 400 mg/l IgG in a 16 h reaction). Following purification using standard techniques, the IgGs were isolated to near homogeneity (Fig. 6a). Under non-reducing conditions, a single band is observed on SDS–PAGE consistent with each correctly assembled full-length IgG. Under reducing conditions, one band for the HC and one for the LC is observed. The non-reduced IgGs were also analyzed by Caliper which reveal the high purity of all the fully assembled IgGs (Fig. 6b). The concentrations of the fully assembled IgGs were also determined by Caliper for subsequent affinity measurements by comparing with a wt trastuzumab IgG standard.

To estimate the affinity, an initial ELISA titration reveals that the IgGs have sub-nanomolar EC_50_ binding to CEA (Fig. 6c). None of the purified IgGs showed any significant binding to the streptavidin control in the ELISA (data not shown). Surface plasmon resonance (SPR) was used to measure the binding kinetics and affinities (Fig. 6d). Streptavidin was immobilized on the chip followed by addition of biotinylated CEA. A dilution series of the IgGs were then injected on the chip to measure the binding affinities (Table I). Binding kinetics and affinities (in the 10–20 nM range) were determined using a bivalent model which fit the data better than a 1 : 1 model as would be expected for bivalent IgGs. All the IgGs also show low nanomolar *K*_D_ affinities to CEA-expressing MKN45 cells, while the trastuzumab wt IgG does not (Fig. 6e). Lastly, we show that the CEA-specific IgGs no longer bind to ErbB2/HER2 overexpressing breast cancer SKBR3 cells (Fig. 6f and Supplementary Fig. S6).

**Table I.** Binding kinetics and affinities determined by SPR for purified IgGs to CEA^a^

IgG	*k*_on_ (M^−1^ s^−1^)	*K*_off_ (s^−1^)	*K*_D_ (nM)
2-B5	5.0 ± 0.9 × 10^5^	8.0 ± 0.8 × 10^−3^	16 ± 1
2-D1	3.4 ± 0.3 × 10^5^	4.4 ± 0.5 × 10^−3^	13 ± 3
2-G3	2.6 ± 0.1 × 10^5^	4.9 ± 0.6 × 10^−3^	19 ± 2
3-E6	2.3 ± 0.1 × 10^5^	3.7 ± 0.5 × 10^−3^	16 ± 1

^a^Values for the first binding event are reported as the mean ± standard deviation of three independent global fits in three different channels for 2-D1, 2-G3 and 3-E6 and two independent channels for 2-B5. A single outlier for 2-B5 was excluded (*Q* = 0.98).

### Construction of a naïve, synthetic Fab LC display library

**Fig. 7.**
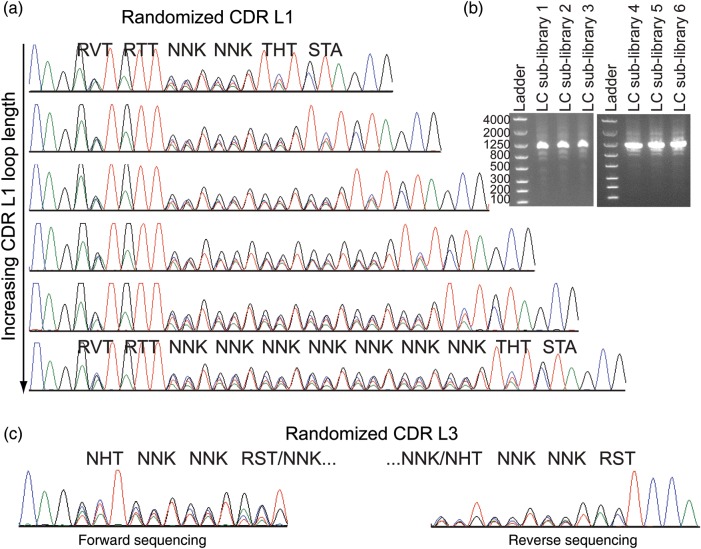
Fab LC library characterization. (**a**) Pooled sequencing of each naïve Fab LC sub-library shows the intended degeneracy and loop lengths in CDR L1. For clarity, only the degenerate codons are labeled for the shortest and longest loop-length sub-libraries. (**b**) The final crude assembled libraries consist of a single band of the desired size on an agarose gel. (The original LC display template is 1076 bps). (**c**) Pooled sequencing of the mixture of CDR L3 loop lengths necessitates sequencing in forward and reverse to confirm the intended randomization since the mixture of different loop lengths shifts the sequencing register. The L3 sequencing is only shown for the shortest L1 sub-library since all libraries show the same results.

The above experiments demonstrate that it is possible to use Fab HC libraries to select novel, functional IgGs *in vitro*, but they do not address whether one can discover leads using a Fab LC library in the same manner. To test this possibility, a Fab LC library was designed based on previously validated LC libraries used in phage display selections (Lee *et al.*, 2004; Bostrom *et al.*, 2009). Replication of the exact Bostrom *et al.* library would require 62 oligos using our previously described protocol (Fig. 2), so the library was simplified to enable library construction with only 20 oligos (Table II and Supplementary Table SIII). Our library included six different loop lengths incorporated into CDR L1 and three different loop lengths in CDR L3. Separate Fab LC sub-libraries with defined CDR L1 loop lengths were constructed. Following overlap assembly PCR, a single dominant band is visible for each sub-library of the expected size (Fig. 7b). Pooled sequencing clearly shows the intended loop-length diversity in CDR L1 for each sub-library (Fig. 7a). The oligos encoding the CDR L3 randomizations were simply mixed in each L1 sub-library to enable simultaneous incorporation of all CDR L3 loop lengths. The mixture of L3 loop lengths in each sub-library complicates analysis by pooled sequencing since each L3 loop-length shifts the sequencing register at the site of loop-length divergence. Sequencing in forward and reverse directions enables reading up to the site of loop-length variability consistent with the intended L3 randomization (Fig. 7c). Pooled sequencing also confirms the intended randomization of L2 (not shown).

**Table II.** Amino acid diversity encoded in the Fab LC library

CDR	Position	Codon	Natural diversity^a^	Designed diversity	Reference
L1	28	RVT	SGDNTR	SNTGDA	None^b^
	29	RTT	IVL	IV	^a^
	30	NNK	SLRGNVTD	SLRGAVTPX	^a^
	30a–e	NNK	SNYGKHD	SLRGAVTPX	^a^
	31	NNK	SNTRDIK	SLRGAVTPX	^a^
	32	THT	YNWFSDR	YFS	^c^
	33	STA	LVFX^d^	LV	^a^
L2	50	KBG	GADWKLES	GAWLSV	^c^
	51	GST	AVGT	AG	^a^
	52	AGT	S	S	^a^
	53	AVC	SNTKRI	SNT	^c^
L3	91	NHT	YSRAGHFLD	STYLFAPVINDH	None^b^
	92	NNK	GYNSDLTHI	SLRGAVTPX^d^	None^b^
	93	NNK	SNQTHGDR	SLRGAVTPX^d^	None^b^
	93a–b	NNK	WSTPLYVAN	SLRGAVTPX^d^	None^b^
	94	RST	TPSWYLFAVG	TSAG	^a^

^a^The codon/diversity was previously reported by Bostrom *et al.* (2009).

^b^The codon/diversity was not derived from any particular reference.

^c^The codon/diversity was previously reported by Lee *et al.* (2004).

^d^X indicates additional amino acids at low percentages.

### Performing a Fab display selection from a naïve Fab LC library

**Fig. 8.**
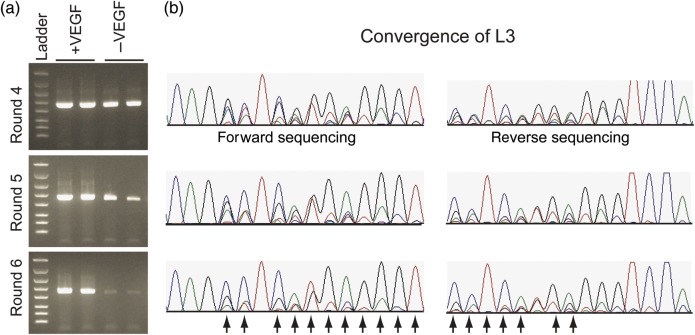
Fab LC selection against VEGF. (**a**) Selections were performed in the presence or absence of VEGF in duplicate. By Round 5, there is a clear difference in the amount of DNA recovered in the presence versus the absence VEGF following RT-PCR. The ladder is the same as in Fig. 2b. (**b**) Pooled sequencing centered on CDR L3 of the output DNA following RT-PCR after each selection round indicates convergence to specific bases or mixtures of bases (arrows) from the original degenerate codons. For brevity, only data for Rounds 4–6 are shown for CDR L3. The other CDRs exhibit similar levels of enrichment (not shown).

Each of the LC sub-libraries was pooled at an equimolar ratio prior to running the selection. For the first selection round, 2 µg of library DNA (∼1.9 × 10^12^ molecules) was used for the cell-free reactions. The LC display library and the constant HC expression DNA templates were mixed at a 1 : 1 stoichiometric ratio throughout the selection against biotinylated VEGF. By round 5, there is a clear increase in the recovery of DNA following RT-PCR in the presence of the VEGF over the no antigen control (Fig. 8a). As with the HC selection, the convergence by pooled sequencing correlates well with the enrichment observed by RT-PCR (Fig. 8b). The selection was carried through six rounds to improve the enrichment of VEGF binders.

### Screening and characterizing IgGs from a Fab LC selection

Unlike the Fab HC, which needs to be reformatted into a full-length HC to screen in an IgG format, the selected LC can be used without modification to screen as IgGs since the Fab and IgG LCs are equivalent. Thus, the selected LCs from Round 6 were cloned directly into a suitable vector for cell-free expression giving a total of 84 LC sequences with 41 unique CDR combinations (Supplementary Fig. S7). Like the Fab HC selection, most of the unique CDR combinations consist of mixtures of the same set of CDRs with 14 unique L1, 14 L2 and 21 L3 CDRs. As with the Fab HC selection each construct has additional mutations outside of the CDRs obtained during the ribosome display selection, so most clones have overall unique sequences even if they have the same set of CDRs. A range of L1 and L3 loop lengths was observed—consistent with the initial library design.

**Fig. 9.**
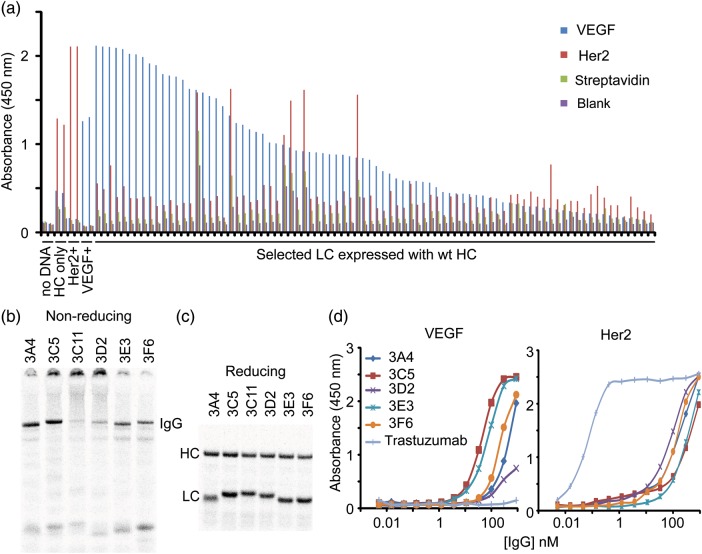
Screening and expression of IgGs with selected LCs. (**a**) An ELISA from all 84 selected LCs was run indicating most clones show specificity for VEGF when expressed with the wt trastuzumab IgG HC. The clones were ranked according to total VEGF signal. Cell-free reactions without DNA (negative control), trastuzumab IgG HC + LC (Her2 positive control), trastuzumab IgG HC only and a mouse anti-VEGF antibody (VEGF positive control) were included on the same ELISA (in duplicate). The negative and positive controls show the expected absence and presence of VEGF-specific or Her2/ErbB2 signal, respectively. The wt trastuzumab IgG HC expressed without the LC shows some specificity for Her2 by itself. (**b**) A non-reducing SDS–PAGE gel shows significant expression of each selected IgG at the correct molecular weight for the fully assembled product. Proteins are labeled with ^14^C-Leu. Aggregates are observed with clones 3C5, 3C11 and 3D2. (**c**) A reducing SDS–PAGE gel shows both the HCs and LCs express well separately. Proteins are also labeled with ^14^C-Leu. (**d**) ELISA titrations using purified and normalized IgGs were performed to assess the relative binding affinities to VEGF and Her2/ErbB2. Most of the IgGs show modest affinities to VEGF and weaker affinities to Her2.

All 84 LC sequences were expressed in small-scale (30 µl) cell-free reactions with the full-length trastuzumab IgG HC to screen directly as IgGs by ELISA (Fig. 9a). The majority of the IgGs with the selected LCs show specificity for the VEGF target though a handful show non-specific binding. Many clones also show binding to the original Her2/ErbB2 antigen albeit at much lower levels. A control experiment in which the trastuzumab HC was expressed alone also showed specificity for Her2/ErbB2. Thus, it was unclear from this ELISA if the apparent retention of binding to Her2/ErbB2 was simply due to an excess of free HC in the crude small-scale cell-free reactions.

To better characterize clones from the selection, six VEGF positive clones covering a range of CDR sequences were chosen for scale-up (20 ml cell-free expressions). Most of the IgGs expressed and assembled at reasonable titers (four of six), but a couple (3C11 and 3D2) showed a propensity to aggregate (Fig. 9b). All HCs and LCs showed approximately the same levels of expression in isolation as assessed by non-reducing gels (Fig. 9c). A total of four of the six IgGs were purified to near homogeneity and a small amount of 3D2 was obtainable (Supplementary Fig. S8a). After purification, the IgG concentrations were normalized and their binding assessed by ELISA except for 3C11, which yielded too little assembled IgG (Fig. 9d). Most of the remaining clones showed between 50 and 100 nM EC_50_ values for VEGF and weaker binding to Her2/ErbB2 except for 3D2, which showed higher affinity for the original Her2/ErbB2 antigen. Overall, the binding affinities are significantly lower than the trastuzumab control for Her2 and weaker than the IgGs from the CEA HC selections. None of the clones showed significant background binding to streptavidin except for 3F6 (Supplementary Fig. S8).

SPR was also used to measure the affinities kinetics of the antigen–antibody interactions (Tables III and IV). The antigens (rhVEGF121 and rhErbB2/Her2-Fc) were directly immobilized on the CM5 chip surface, and a dilution series of the antibody variants were injected over the antigen surface. As positive controls, the affinities of the trastuzumab IgG and ranibizumab Fab (both expressed in our cell-free extract) were also examined against both Her2/ErbB2 and VEGF. The trastuzumab IgG showed the expected binding for Her2/ErbB2 antigen, but did not show any measurable binding to VEGF (not shown). Similarly, the ranibizumab Fab showed the expected binding to VEGF, but did not show any measurable binding to Her2/ErbB2 (not shown). As expected for bivalent antibodies, the data for the IgGs from the Fab LC selection against VEGF and the trastuzumab IgG control fit better with a bivalent analyte model than a 1 : 1 model, while the data for ranibizumab Fab was fit using a 1 : 1 binding model. All the selected IgGs showed some measurable binding to both VEGF and Her2/ErbB2, except 3F6 which showed non-specific binding to the chip, so the affinity for this clone could not be measured. Only the 3E3 IgG exhibited slow-on/slow-off kinetics in binding to VEGF, while the other variants tested had relatively faster off-rates. Consistent with the ELISA titrations, the 3E3 IgG also showed the highest affinity to both VEGF and Her2/ErbB2 and 3D2 showed weaker binding to VEGF and stronger binding to Her2/ErbB2. Interestingly, the relative affinity of 3C5 for VEGF appears to be weaker by SPR than ELISA. Overall, the SPR experiments are generally consistent with the ELISA titrations in that the IgGs from the LC selection show weaker binding affinities than IgGs from the HC selection for their respective antigens.

**Table III.** Binding kinetics and affinities determined by SPR for purified IgGs to VEGF

IgG	*k*_on_ (M^−1^ s^−1^)	*k*_off_ (s^−1^)	*K*_D_ (nM)
3A4	2.00 × 10^4^	1.13 × 10^−2^	567
3C5	3.23 × 10^4^	1.55 × 10^−2^	479
3D2	5.02 × 10^4^	5.06 × 10^−2^	1010
3E3	2.09 × 10^4^	1.45 × 10^−3^	69.3

**Table IV.** Binding kinetics and affinities determined by SPR for purified IgGs to Her2/ErbB2

IgG	*k*_on_ (M^−1^ s^−1^)	*k*_off_ (s^−1^)	*K*_D_ (nM)
3A4	1.20 × 10^4^	1.12 × 10^−2^	933
3C5	9.50 × 10^3^	2.29 × 10^−3^	241
3D2	5.15 × 10^4^	1.71 × 10^−3^	33.2
3E3	1.70 × 10^4^	2.82 × 10^−3^	166

## Discussion

We present a simple approach for the *in vitro* selection of multi-chain proteins. Specifically, we used standard ribosome display protocols to perform selections for heterodimeric Fabs. Two-chain Fab display works by circumventing the presumed requirement of maintaining the genotype–phenotype connection between all the protein fragments in the selection (Sumida *et al.*, 2009; Ponsel *et al.*, 2011). We show that retaining the genotype of a single protein chain is sufficient for the selection of novel multi-chain proteins. This approach should apply generally to other multi-chain proteins besides antibodies and other *in vitro* selection technologies like mRNA (Roberts and Szostak, 1997), CIS (Odegrip *et al.*, 2004) or CAD display (Reiersen *et al.*, 2005). Though our approach may limit library selections to a single chain at one time, it also enables the selection of significantly larger libraries of multi-chain proteins than other common approaches. Moreover, selections performed with Fab HC libraries alone suggest a straightforward route to discovering bi-specific antibodies which share a common LC. Two Fab HC leads could simply be reformatted to heterodimeric IgGs using CH3 knob-hole or complementary charge variants (Ridgway *et al.*, 1996; Merchant *et al.*, 1998; Gunasekaran *et al.*, 2010). Selections performed exclusively with a library on a single chain seem likely to cause certain leads to be missed since the non-randomized chain might be sub-optimal for binding. This does not seem to be a significant hindrance for finding high-affinity leads as we present here using ribosome display with the Fab HC library or in phage display where libraries built on separate chains have been used (Lee *et al.*, 2004; Bostrom *et al.*, 2009).

Though selections with both the HC and LC libraries led to novel binders, the HC library led to higher affinity and better behaved IgGs against CEA than the selection with the LC library against VEGF. Differences in library design and the antigen target between the selections might explain the different results. On the other hand, it is not surprising that the HC selection performed better than the LC selection given that H3 is generally recognized to be the most important CDR for antigen binding and the most variable in natural systems (Collis *et al.*, 2003; Zemlin *et al.*, 2003). Moreover, the HC often completely dominates interactions with antigens (Muller *et al.*, 1998) and some species have functional antibodies consisting of only HCs without LCs (Muyldermans, 2013). This also rationalizes why the IgGs from our LC selections still retained binding to the original Her2/Erb2 antigen albeit at weaker affinities. Thus, focusing Fab library selections on the HC appears better than the LC. For a given target it seems reasonable to first use a HC library and then a LC library for affinity maturation as has been recommended previously (Lee *et al.*, 2004). Nonetheless, improvements in library design on the LC might also increase the affinities and biophysical behavior of the resulting IgGs from Fab LC selections.

As most current antibody therapeutics are IgGs, one might consider the direct display of whole IgGs using an *in vitro* selection platform. Indeed, we have shown here and previously (Yin *et al.*, 2012) that it is possible to produce IgGs in cell-free expression systems used for *in vitro* selection platforms. However, IgG display would be complicated by the antibody's structure. Using a single expression and display template for the HC and LC should produce heterodimers of heterodimers, i.e. two different HC/LC dimers could dimerize. In this format, only a single HC/LC pair would need to bind to the antigen for the IgG to survive the selection and another non-binding HC/LC pair could survive by dimerization. In theory, monovalent IgG display should be possible by using an excess of two constant HC and LC expression templates and a library display template at a much lower ratio. This would lead to a majority of free IgGs unconnected to the display transcript, but a statistical majority of the displayed IgGs would maintain linkage to a single library genotype. This would consume cell-free expression resources, however, and limit the effective library size.

*In vitro* Fab display does not suffer from the structural complications of IgG display. Moreover, we did not experience any difficulty converting Fabs from our selection to IgGs for immediate expression, screening and characterization. A recent study compared the efficiency of converting semi-synthetic scFv and human-derived Fab libraries to IgGs (Chan *et al.*, 2011). They found several of their scFvs had lower binding specificity when converted to IgGs and a large number of the semi-synthetic CDRs contained amber stop codons and glycosylation sites *following* selection by phage display. This complicated scFv conversion to IgGs, so they argue for the use of naturally derived versus synthetic libraries. We did not observe any amber stop codons in the CDRs from our synthetic Fab libraries presumably due to minimal amber suppression in the cell-free extract used during selections. Also, glycosylation sites in the CDRs have no detrimental effects if aglycosylated IgGs are expressed in cell-free extract. Though scFv to IgG conversion often works well (Tang *et al.*, 2007), the ease at which Chan *et al.* converted their Fabs to IgGs suggests starting from Fabs is preferable to scFvs regardless if the initial library is of synthetic or natural origin. Thus, our Fab display system in conjunction with optimized cell-free expression for the production of IgGs constitutes a novel platform for the rapid discovery of synthetic or natural antibodies.

## Supplementary data

Supplementary data are available at *PEDS* online.

## Funding

This work was privately funded. Funding to pay the Open Access publication charges for this article was provided by Sutro Biopharma, Inc.

## Supplementary Material

Supplementary Data
