# Supplementary material for: In vitro Fab display: a cell-free system for IgG discovery
Source: Protein Eng Des Sel. 2014 Feb 28;27(4):97–109. doi: 10.1093/protein/gzu002 (PMC3966677; doi:10.1093/protein/gzu002)
Supplement: Supplementary Data [file supp_27_4_97__index.html]

In vitro Fab display: a cell-free system for IgG discovery — In vitro Fab display: a cell-free system for IgG discovery — Supplementary Data 

# *In vitro* Fab display: a cell-free system for IgG discovery

## Supplementary Data

Supplementary Data

**Files in this Data Supplement:**

- Supplementary Figure 1 - pdf file
- Supplementary Figure 2 - pdf file
- Supplementary Figure 3 - pdf file
- Supplementary Figure 4 - pdf file
- Supplementary Figure 5 - pdf file
- Supplementary Figure 6 - pdf file
- Supplementary Figure 7 - pdf file
- Supplementary Figure 8 - pdf file
- Supplementary Data - Docx file
- Supplementary Tables - docx file
