# Supplementary figures and images for: In vitro Fab display: a cell-free system for IgG discovery
Source: Protein Eng Des Sel. 2014 Feb 28;27(4):97–109. doi: 10.1093/protein/gzu002 (PMC3966677; doi:10.1093/protein/gzu002)

Figure S1

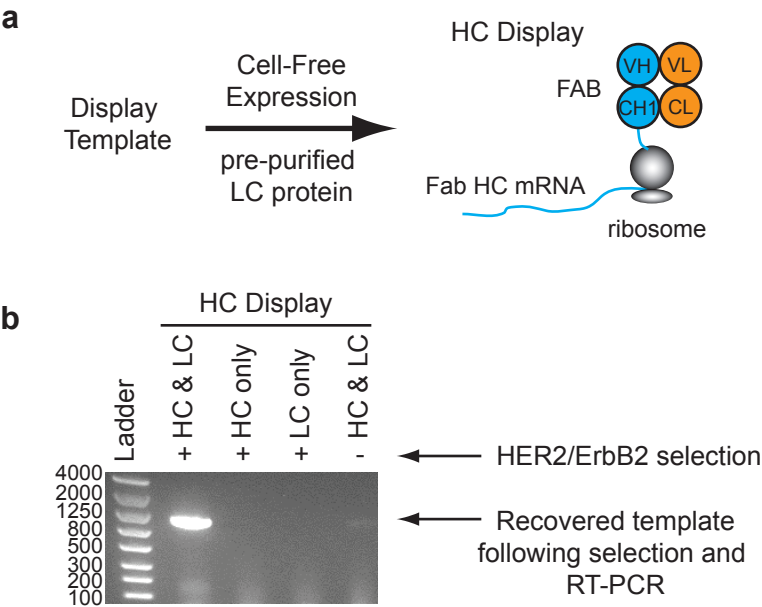

Supplement: Supplementary Data [file supp_gzu002_gzu002supp_fig1.pdf]

Figure S2

a

Randomized CDR1

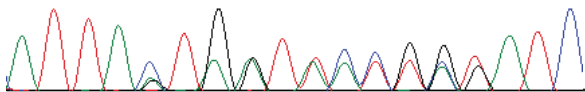

b

Randomized CDR2

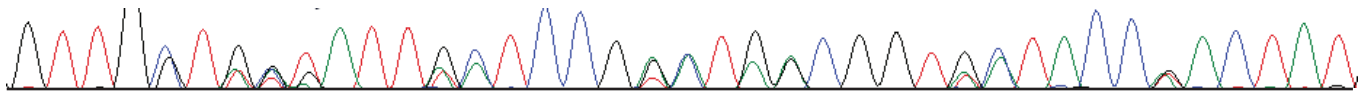

Supplement: Supplementary Data [file supp_gzu002_gzu002supp_fig2.pdf]

**Figure S3**

**a**

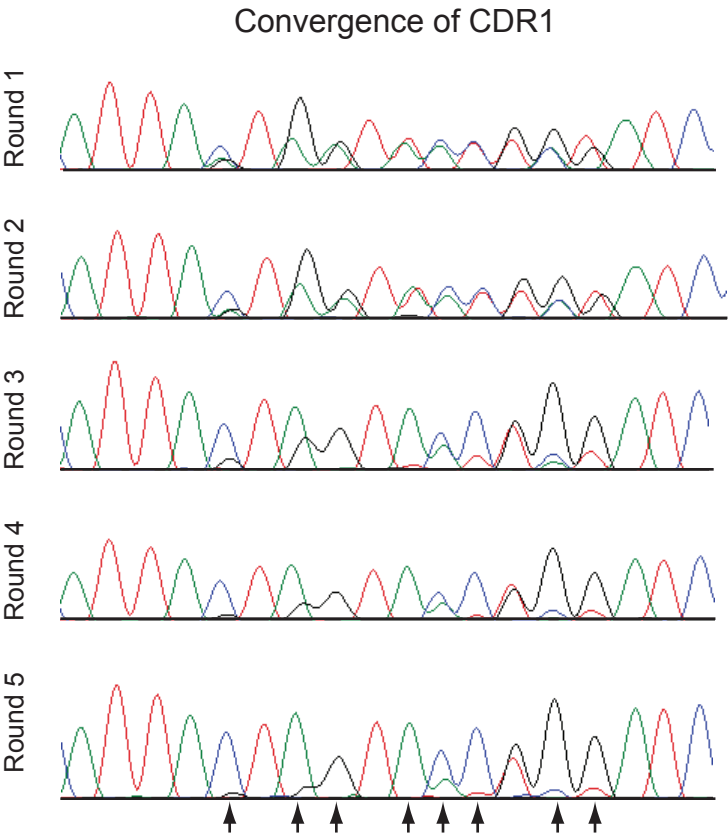

**b**

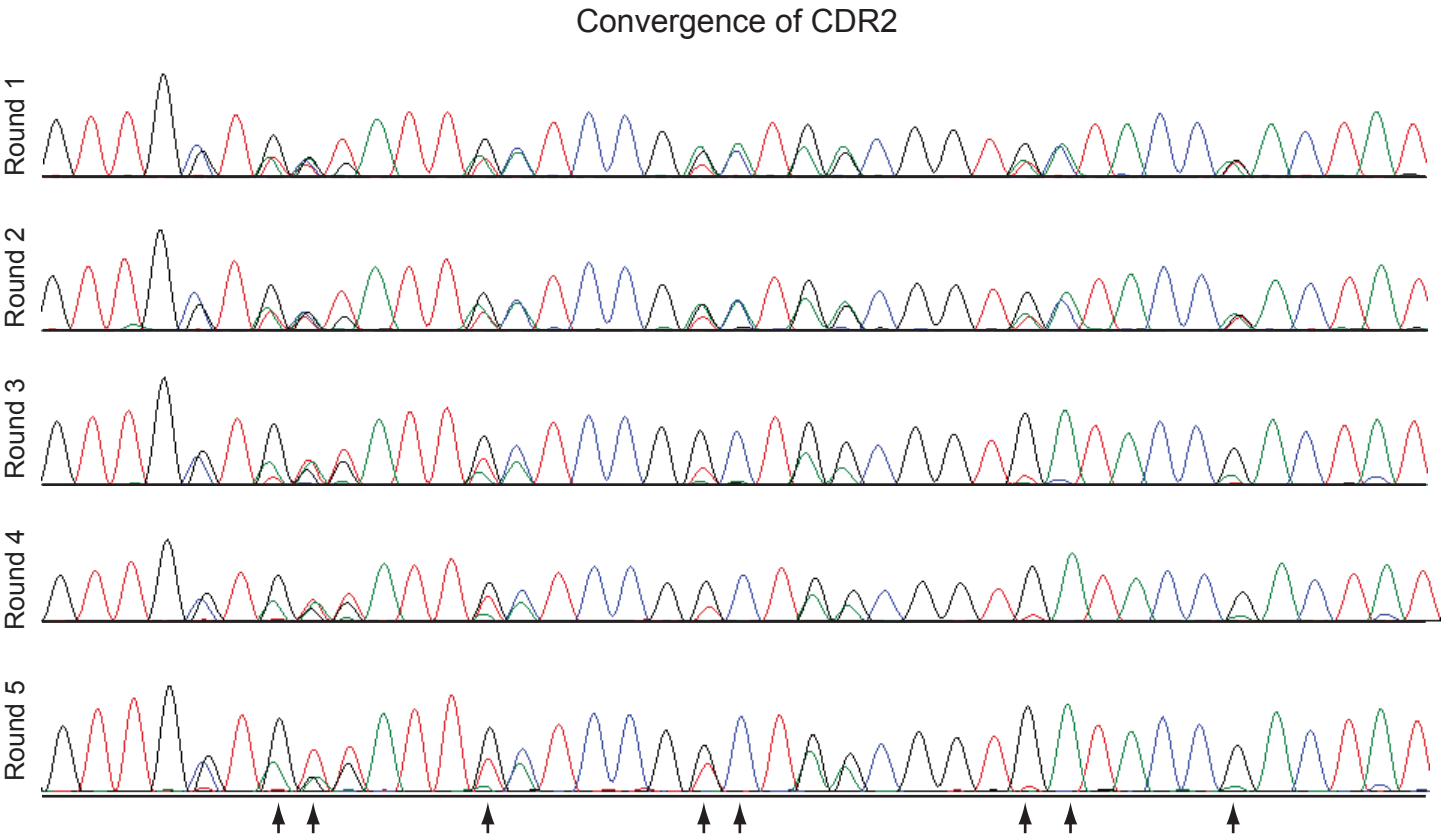

Supplement: Supplementary Data [file supp_gzu002_gzu002supp_fig3.pdf]

Figure S5

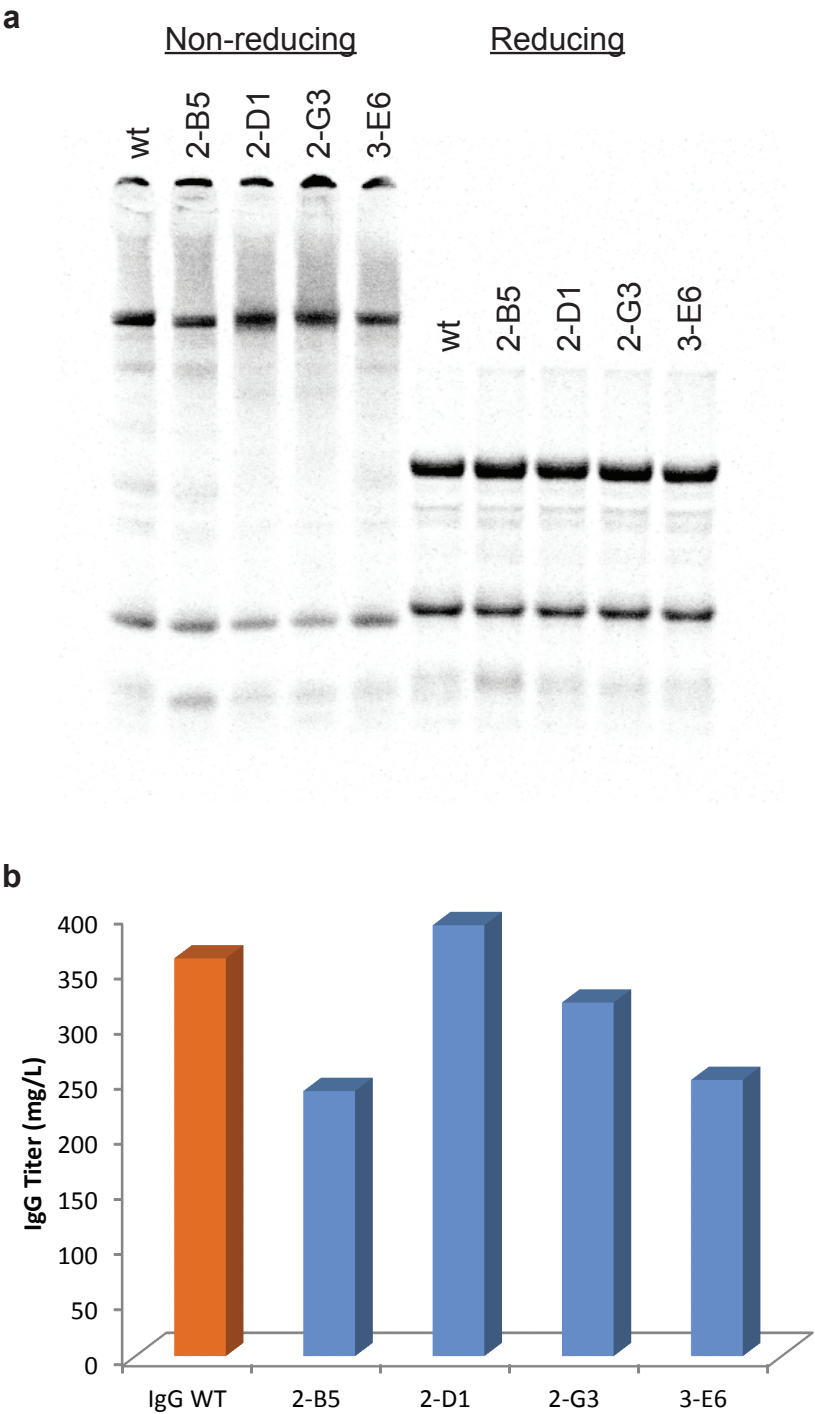

Supplement: Supplementary Data [file supp_gzu002_gzu002supp_fig5.pdf]

Figure S6

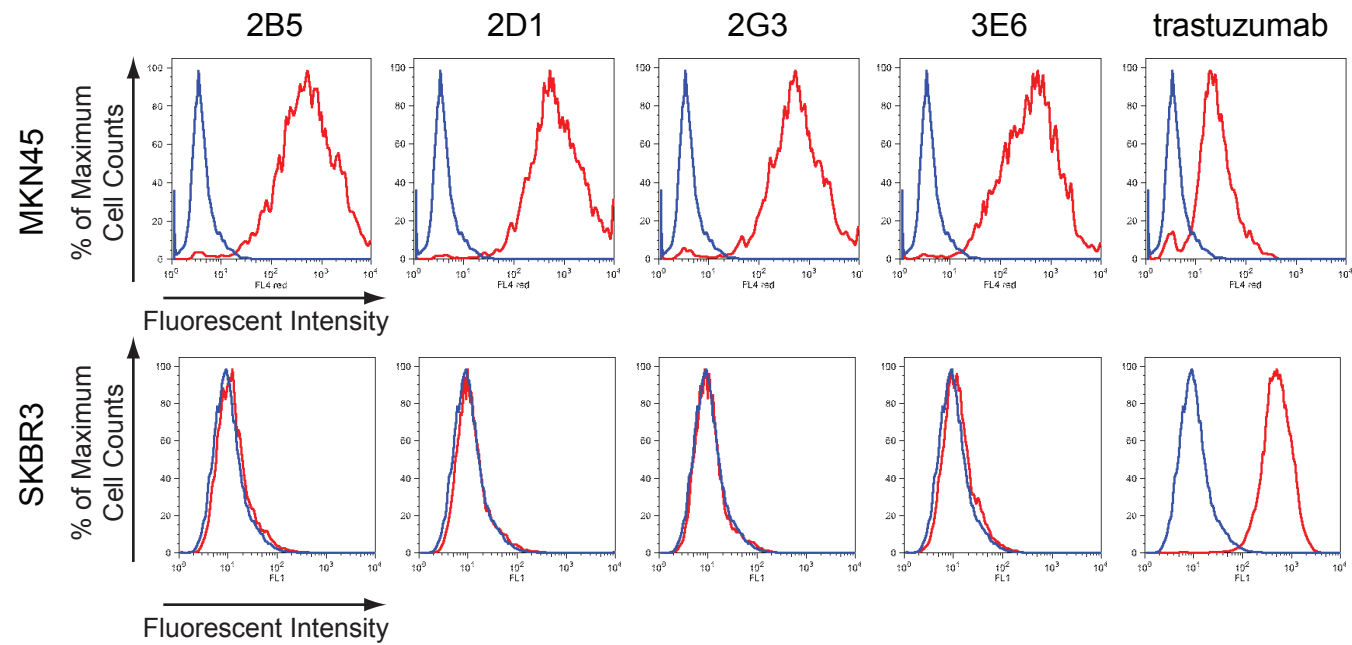

Supplement: Supplementary Data [file supp_gzu002_gzu002supp_fig6.pdf]

Supplemental Figure S8

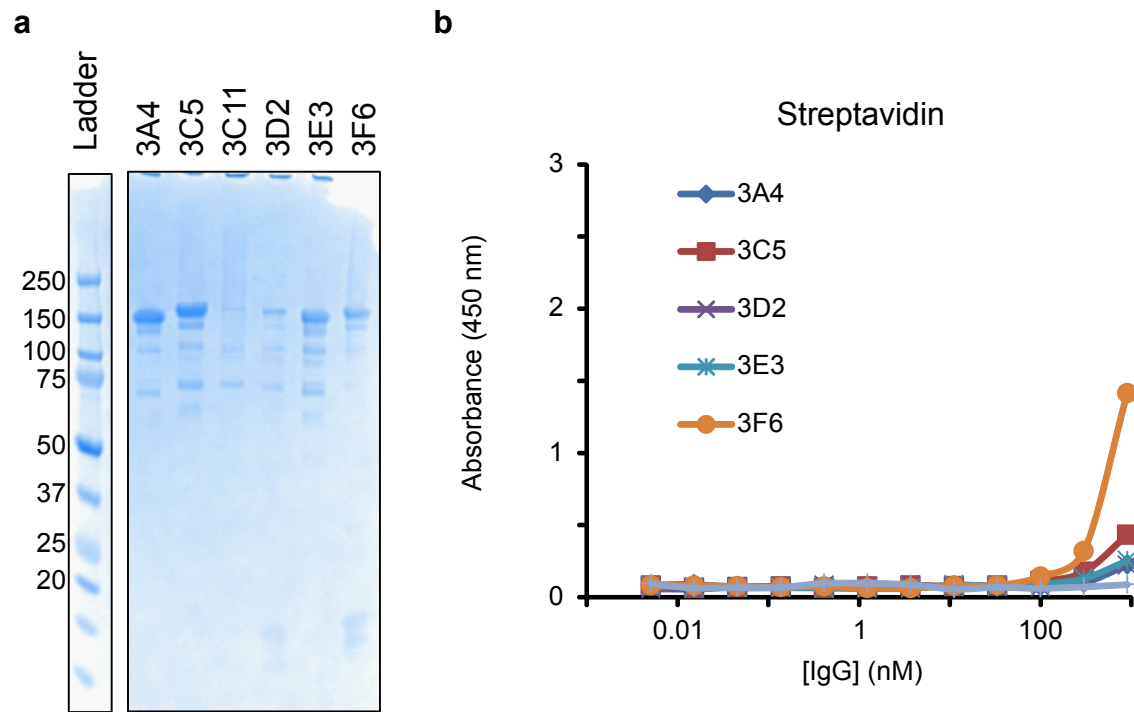

Supplement: Supplementary Data [file supp_gzu002_gzu002supp_fig8.pdf]
