# Supplementary material for: In vitro Fab display: a cell-free system for IgG discovery
Source: Protein Eng Des Sel. 2014 Feb 28;27(4):97–109. doi: 10.1093/protein/gzu002 (PMC3966677; doi:10.1093/protein/gzu002)
Supplement: Supplementary Data [file supp_gzu002_gzu002supp_fig4.pdf]

Figure S4

Total	CDR H1	CDR H2	CDR H3	Representative Clone
10	T S T G	G V I A P S N G D T D	R W V W G L V V F	2-B5
4	T S N W	A R I Y P A G G D T D	S F A P S V A W M	
3	T S T G	A R I Y P A G G D T D	S F A P S V A W M	2-G3
2	T S T G	G V I A P S N G D T D	Q F C P W C P Y M	
1	N S S D	G R I D P Y S G D T D	T R V H K W S T M	
1	S S T G	A R I Y P A G G D T D	S F A P S V A W M	
1	S S T W	A R I Y P A G G D T D	R W V W G L V V F	2-D1
1	S S T W	A R I Y P A G G D T D	S F A P S V E W M	
1	S S T W	G D I T P A G G D T N	Q F C P W C P Y M	
1	S S T W	G D I T P A G G D T N	R W V W G L V V F	
1	S S T W	G D I T P A G G D T N	S F A P S V A W M	
1	T S A G	G V I A P S N G D T D	S F A P S V A W M	
1	T S N S	A E I S P A S G Y T D	N W A V G M K V M	
1	T S N W	A R I Y P A G G D T D	N W A V G M K V M	
1	T S N W	A R I Y P A G G D T D	R W V W G L V V F	
1	T S N W	A R I Y P A G G G T D	S F A P S V A W M	
1	T S N W	G V I A P S N G D T D	R W V W G L V V F	
1	T S T G	A G I A P A G G T T Y	N W A V G M K V M	
1	T S T G	A R I Y P A G G D T D	Q F C P W C P Y M	3-E6
1	T S T G	G R I D P A D G S T D	V W V P T M H V M	
1	T S T G	G V I A P S N G D T D	N W A V G M K V M	
1	T S T G	G V I A P S N G D T D	P F A P S V A W M	
1	T S T G	G V I D P S N G D T D	R W V W G L V V F	
1	T S T W	A I I A P A D G D T D	R W V W G L V V F	
