# Supplementary material for: In vitro Fab display: a cell-free system for IgG discovery
Source: Protein Eng Des Sel. 2014 Feb 28;27(4):97–109. doi: 10.1093/protein/gzu002 (PMC3966677; doi:10.1093/protein/gzu002)
Supplement: Supplementary Data [file supp_gzu002_gzu002supp_fig7.pdf]

Figure S7

Total	L1	L2	L3	Representaitve Clone
32	: : : : : : G V V I V	S A S N	: : P H E G	3A4
5	N V : : : : G : W F V	W G S T	: T W Y : T	
4	N I S H K H A H : F L	G A S N	: : D L S S	3C11
3	A I : : : : R V P F V	W G S S	L : D V G G	3F6
2	: : : : : : G V V I V	S A S N	: : D L S S	
2	A I T H R S A H : F L	G A S N	: : D L S S	3C5
2	A I T H R S A H : F L	G A S N	: : P H E G	
1	: : : : : : G V A I V	S A S S	P T G : : T	3E3
1	: : : : : : G V V I V	G A S N	: : P H E G	
1	: : : : : : G V V I V	S A S N	: D A W : G	
1	: : : : : : G V V I V	S A S N	: P S : S A	
1	: : : : : : G V V I V	S A S N	: S A : V T	
1	: : : : : : G V V I V	S A S N	: T W Y : T	
1	: : : : : : G V V I V	S A S N	I : : T S S	
1	: : : : : : G V V I V	S A S N	P G G : : A	
1	: : : : : : G V V I V	S A S N	P T G : : T	
1	: : : : : : G V V I V	S A S N	V R V P M T	
1	: : : : : : G V V I V	W G S N	: : P H E G	
1	: : : : : G V V I A :	G A S N	: V R W : G	
1	: : G V V I G : : : :	S A S N	: : P H E G	
1	A I : : : : R V P F V	G A S T	N : A W : G	
1	A I : : : : R V P L V	W G S S	L : D V G G	
1	A I T H R S A H : F L	G A S N	: : V R A S	
1	A I T H R S A H : F L	G G S N	: : D L S S	
1	A I T H R S A H : F L	G G S N	: : P H E G	
1	A I T H R S A H : F L	G G S N	L : D V G G	
1	A V : : : : : Q : I V	W A S T	P G G : : A	
1	D V : : : : : Y R P V	A G S T	: : P H E G	
1	D V : : : : : Y R S V	A A S N	: : D L S S	
1	D V : : : : : Y R S V	A G I S	V R V P M T	
1	D V : : : : : Y R S V	A G S S	: : V R A G	
1	D V : : : : : Y R S V	A G S S	I : : R Q G	
1	D V : : : : : Y R S V	A G S S	I : : T S S	
1	D V : : : : : Y R S V	A G S S	I : A : Q A	3D2
1	G V : R T G T P V Y V	W A S S	L : W M T S	
1	N I S H K H A H : F L	G A S N	: : P H E G	
1	N I S H K H A H : F L	G A S N	L : D V G G	
1	N V : : : : G : W F V	G A S N	Y : N F : T	
1	N V : : : : G : W F V	W G S T	: : D L S S	
1	N V : : : : G : W F V	W G S T	: T W Y : N	
1	N V : G W A G Q : F L	G G S N	: : P H E G	
