# Supplementary material for: In vitro Fab display: a cell-free system for IgG discovery
Source: Protein Eng Des Sel. 2014 Feb 28;27(4):97–109. doi: 10.1093/protein/gzu002 (PMC3966677; doi:10.1093/protein/gzu002)
Supplement: Supplementary Data [file supp_gzu002_gzu002supp.docx]

**Fig. S1. Conceptual Overview of Fab Display Using Pre-Purified Protein.** (a) A mixture of the display template and pre-purified protein can also be used to display functional heterodimeric proteins on ribosomes provided one domain can be expressed and purified in soluble form. In the case of Fab domains, the LC can be pre-expressed, purified, and added to the cell-free mixture to produce functional Fabs. This obviates the need to co-express the LC protein simultaneously with the HC. (b) Consistent with the co-expression format shown in Figure 1, *both* the HC and LC trastuzumab proteins are required for significant recovery of the template following selection. This is also consistent with the assembly of functional heterodimeric Fabs on stalled ribosomes.

**Fig. S2. Characterization of CDR1 and CDR2 from the Naïve Library.** Pooled sequencing of the final library is consistent with the intended randomization at (a) CDR1 and (b) CDR2.

**Fig. S3. Analysis of CDR1 and CDR2 During Selection.** Pooled sequencing after each round of selection following RT-PCR suggests convergence of both (a) CDR1 and (b) CDR2. The arrows indicate specific bases which have converged.

**Fig S4. CDR Sequences from Fab HC Selections.**  The total number of times the unique CDR sequences were isolated is shown on the left. Representative clones chosen for scale-up and further characterization are indicated on the right.

**Fig. S5. Large-scale Expression of IgGs Analyzed by ^14^C Autoradiography.** (a) An aliquot of 100 µL of the 20 mL cell-free reactions were removed and expressed in the presence of ^14^C-Leu. The samples were run either reduced or non-reduced on SDS-PAGE. (b) The yields of the anti-CEA IgGs were comparable to the wt trastuzumab IgG.

**Fig. S6. FACS Histograms for CEA-binding IgGs.** The selected IgGs clearly show strong specific binding to MKN45 (high CEA expression) over SKBR3 (low CEA expression). MKN45 expresses Her2/ErbB2 at low levels explaining why trastuzumab binds to these cells at lower levels. The secondary antibody is shown in blue and the primary + secondary antibody is shown in red.

**Fig S7. CDR Sequences from Fab LC Selections.**  The total number of times the unique CDR sequences were isolated is shown on the left. Representative clones chosen for scale-up and further characterization are indicated on the right.

**Fig S8. Additional characterization of IgGs from Fab LC selections.** (a) All of the IgGs were able to be purified except 3C11. The yield for 3D2 was relatively low. (b) Background binding to streptavidin was assessed by ELISA for the purified IgGs. None of the purified IgGs showed significant background binding to streptavidin except for 3F6.
