# Supplementary material for: In vitro Fab display: a cell-free system for IgG discovery
Source: Protein Eng Des Sel. 2014 Feb 28;27(4):97–109. doi: 10.1093/protein/gzu002 (PMC3966677; doi:10.1093/protein/gzu002)
Supplement: Supplementary Data [file supp_gzu002_gzu002supp_tables.docx]

**Supplementary Table 1. Primers for PCR and RT-PCR***

| Name | Sequence (5’ to 3’) |
| --- | --- |
| HER2_FAB_HC_STOP! RP | TTA TTA ACA ACA AGA TTT CGG CTC CAC CT |
| HER2_FAB_LC_STOP! RP | TTA TTA GCA CTC ACC GCG GTT AAA |
| FAB FP | ATG GAA GTT CAA TTA GTA GAA AGC GGC G |
| FAB RP | ACA AGA TTT CGG CTC CAC CTT CTT G |
| Fc FP | CAA GAA GGT GGA GCC GAA ATC TTG T |
| Fc RP | CTT ACC CGG GGA CAG GGA CAA G |
| HC FP | TAT ACA TAT GGA AGT TCA ATT AGT AGA AAG CGG CG |
| HC RP | TTT TGT CGA CCT TAC CCG GGG ACA GGG ACA AG |
| Univ TolA RP | TTC AGT TGC CGC TTT CTT TC |
| HER2 HCFAB Inner FP | GAG ATA TAT CCA TGG AAG TTC AAT TAG TAG AAA GC |
| HER2 HCFAB Inner RP | GAG GCG ATA TAA AGC TTA CAA GAT TTC GG |
| HER2 LCFAB Inner FP | ATA TCC ATG GAT ATT CAA ATG ACT CA |
| HER2 LCFAB Inner RP | CGA TAT AAA GCT TGC ACT CAC C |
| FabLC CenterSeq FP | GTA TCA ACA GAA ACC GGG TAA A |
| FabLC CenterSeq RP | TGT TGA CAA TAG TAG GTC GCA AA |
| TolA Internal RP | CTT TGG CGG CTT CTG CTT |
| BsaI HERLC pYD FP | cccccggtctcctatggatattcaaatgactcaaagcccg |
| BsaI HERLC pYD RP | cccccggtctctcgacttagcactcaccgcggttaaaacttttagtg |

*T7B and TolAk primers have been previously reported by Dreier and Plückthun, *Methods Mol. Biol.* **687**, 283-306, (2011).

**Supplementary Table 2. Primers for HC library construction**

| Name | Sequence (5’ to 3’) |
| --- | --- |
| H1a For | cgcggcaagcggttttaacattAVTRRTWMYKMTatccactgggtgcgtcaagcac |
| H1a Rev | gtgcttgacgcacccagtggatAKMRKWAYYABTaatgttaaaaccgcttgccgcg |
| H1b For | cgcggcaagcggttttaacattAVTRRTWMYKGGatccactgggtgcgtcaagcac |
| H1b Rev | gtgcttgacgcacccagtggatCCMRKWAYYABTaatgttaaaaccgcttgccgcg |
| H2a For | gggtaagggcctggaatgggttGSTDGGattDMTccgDMTRRCggtDMTaccDACtatgcggatagcgtgaaaggcc |
| H2a Rev | ggcctttcacgctatccgcataGTHggtAKHaccGYYAKHcggAKHaatCCHASCaacccattccaggcccttaccc |
| H2b For | gggtaagggcctggaatgggttGSTDHTattDMTccgDMTRRCggtDMTaccDACtatgcggatagcgtgaaaggcc |
| H2b Rev | ggcctttcacgctatccgcataGTHggtAKHaccGYYAKHcggAKHaatADHASCaacccattccaggcccttaccc |
| H2c For | gggtaagggcctggaatgggttGSTGAAattDMTccgDMTRRCggtDMTaccDACtatgcggatagcgtgaaaggcc |
| H2c Rev | ggcctttcacgctatccgcataGTHggtAKHaccGYYAKHcggAKHaatTTCASCaacccattccaggcccttaccc |
| H3a For | gcagaggacactgccgtctactattgtGCGcgcNNKNNKNNKNNKNNKNNKNNKNNKatggactactggggccagggtac |
| H3a Rev | gtaccctggccccagtagtccatMNNMNNMNNMNNMNNMNNMNNMNNgcgCGCacaatagtagacggcagtgtcctctgc |
| H3b For | gcagaggacactgccgtctactattgtGCGcgcNNKNNKNNKNNKNNKNNKNNKNNKTTTgactactggggccagggtacgc |
| H3b Rev | gcgtaccctggccccagtagtcAAAMNNMNNMNNMNNMNNMNNMNNMNNgcgCGCacaatagtagacggcagtgtcctctgc |

**Supplementary Table 3. Primers for LC library construction**

| Name | Sequence (5’ to 3’) |
| --- | --- |
| CDR1-0 F | TACCTGCCGTGCGAGCCAGRVTRTTNNKNNKTHTSTAGCGTGGTATCAACAGAAACCGGG |
| CDR1-0 R | CCCGGTTTCTGTTGATACCACGCTASADAMNNMNNAAYABYCTGGCTCGCACGGCAGGTA |
| CDR1-1 F | TACCTGCCGTGCGAGCCAGRVTRTTNNKNNKNNKTHTSTAGCGTGGTATCAACAGAAACCGGG |
| CDR1-1 R | CCCGGTTTCTGTTGATACCACGCTASADAMNNMNNMNNAAYABYCTGGCTCGCACGGCAGGTA |
| CDR1-2 F | TACCTGCCGTGCGAGCCAGRVTRTTNNKNNKNNKNNKTHTSTAGCGTGGTATCAACAGAAACCGGG |
| CDR1-2 R | CCCGGTTTCTGTTGATACCACGCTASADAMNNMNNMNNMNNAAYABYCTGGCTCGCACGGCAGGTA |
| CDR1-3 F | TACCTGCCGTGCGAGCCAGRVTRTTNNKNNKNNKNNKNNKTHTSTAGCGTGGTATCAACAGAAACCGGG |
| CDR1-3 R | CCCGGTTTCTGTTGATACCACGCTASADAMNNMNNMNNMNNMNNAAYABYCTGGCTCGCACGGCAGGTA |
| CDR1-4 F | TACCTGCCGTGCGAGCCAGRVTRTTNNKNNKNNKNNKNNKNNKTHTSTAGCGTGGTATCAACAGAAACCGGG |
| CDR1-4 R | CCCGGTTTCTGTTGATACCACGCTASADAMNNMNNMNNMNNMNNMNNAAYABYCTGGCTCGCACGGCAGGTA |
| CDR1-5 F | TACCTGCCGTGCGAGCCAGRVTRTTNNKNNKNNKNNKNNKNNKNNKTHTSTAGCGTGGTATCAACAGAAACCGGG |
| CDR1-5 R | CCCGGTTTCTGTTGATACCACGCTASADAMNNMNNMNNMNNMNNMNNMNNAAYABYCTGGCTCGCACGGCAGGTA |
| CDR2 F | AAGCCCCGAAGCTGCTGATCTACKBGGSTAGTAVCTTGTATAGCGGTGTTCCGAGCC |
| CDR2 R | GGCTCGGAACACCGCTATACAAGBTACTASCCVMGTAGATCAGCAGCTTCGGGGCTT |
| CDR3-0 F | GAAGATTTTGCGACCTACTATTGTCAACAGNHTNNKNNKRSTCCACCGACGTTCGGCCAG |
| CDR3-0 R | CTGGCCGAACGTCGGTGGASYMNNMNNADNCTGTTGACAATAGTAGGTCGCAAAATCTTC |
| CDR3-1 F | GAAGATTTTGCGACCTACTATTGTCAACAGNHTNNKNNKNNKRSTCCACCGACGTTCGGCCAG |
| CDR3-1 R | CTGGCCGAACGTCGGTGGASYMNNMNNMNNADNCTGTTGACAATAGTAGGTCGCAAAATCTTC |
| CDR3-2 F | GAAGATTTTGCGACCTACTATTGTCAACAGNHTNNKNNKNNKNNKRSTCCACCGACGTTCGGCCAG |
| CDR3-2 R | CTGGCCGAACGTCGGTGGASYMNNMNNMNNMNNADNCTGTTGACAATAGTAGGTCGCAAAATCTTC |
